# *Lutzomyia longipalpis* Saliva Drives Interleukin-17-Induced Neutrophil Recruitment Favoring *Leishmania infantum* Infection

**DOI:** 10.3389/fmicb.2018.00881

**Published:** 2018-05-08

**Authors:** Clarissa R. Teixeira, Claire da S. Santos, Deboraci B. Prates, Rafael T. dos Santos, Théo Araújo-Santos, Sebastião M. de Souza-Neto, Valéria M. Borges, Manoel Barral-Netto, Cláudia I. Brodskyn

**Affiliations:** ^1^Fundação Oswaldo Cruz, Teresina, Brazil; ^2^Instituto Gonçalo Moniz, Fundação Oswaldo Cruz, Salvador, Brazil; ^3^Instituto de Ciências da Saúde da Universidade Federal da Bahia, Departamentos de Biomorfologia e Biointeração, Salvador, Brazil; ^4^Centro de Ciências Biológicas e Saúde, Universidade Federal do Oeste da Bahia, Barreiras, Brazil; ^5^Faculdade de Ciências da Saúde, Universidade Federal da Grande Dourados, Dourados, Brazil; ^6^Faculdade de Medicina da Universidade Federal da Bahia, Departamento de Patologia e Medicina Legal, Salvador, Brazil; ^7^Instituto de Investigação em Imunologia, iii-INCT, São Paulo, Brazil

**Keywords:** *Lutzomyia longipalpis*, sand fly saliva, *Leishmania infantum*, IL-17, neutrophils, macrophages

## Abstract

During bloodfeeding, the presence of sand fly saliva in the hemorrhagic pool where *Leishmania* is also inoculated modulates the development of host immune mechanisms creating a favorable environment for disease progression. To date, information obtained through experimental models suggests that sand fly saliva induces cellular recruitment and modulates production of eicosanoids. However, the effect of sand fly saliva in the different steps of the inflammatory response triggered by *Leishmania* remains undefined. Here we further investigate if interaction of *Lutzomyia longipalpis* salivary gland sonicate (SGS) with different host cells present during the initial inflammatory events regulate *Leishmania infantum* infectivity. Initially, we observed that incubation of human peripheral blood mononuclear cells (PBMC) with *Lu. longipalpis* SGS in the presence of *L. infantum* significantly increased IL-10 but did not alter expression of IFN-γ and TNF-α by CD4^+^ T cells induced by the parasite alone. Interestingly, incubation of PBMC with *Lu. longipalpis* SGS alone or in the presence of *L. infantum* resulted in increased IL-17 production. The presence of IL-17 is related to neutrophil recruitment and plays an important role at the site of infection. Here, we also observed increased migration of neutrophil using an *in vitro* chemotactic assay following incubation with supernatants from PBMC stimulated with *L. infantum* and *Lu. longipalpis* SGS. Neutrophil migration was abrogated following neutralization of IL-17 with specific antibodies. Moreover, culture of human neutrophils with *L. infantum* in the presence of *Lu. longipalpis* SGS promoted neutrophil apoptosis resulting in increased parasite viability. Neutrophils operate as the first line of defense in the early stages of infection and later interact with different cells, such as macrophages. The crosstalk between neutrophils and macrophages is critical to determine the type of specific immune response that will develop. Here, we observed that co-culture of human macrophages with autologous neutrophils previously infected in the presence of *Lu. longipalpis* SGS resulted in a higher infection rate, accompanied by increased production of TGF-β and PGE_2_. Our results provide new insight into the contribution of *Lu. longipalpis* SGS to *L. infantum*-induced regulation of important inflammatory events, creating a favorable environment for parasite survival inside different host cells.

## Introduction

*Leishmania* transmission occurs through the bite of infected female sand flies. Bloodfeeding causes tissue damage creating a hemorrhagic pool resulting from probing and destruction of small capillaries. In this environment *Leishmania* and saliva interact with different host cells including peripheral blood and resident cells in the skin (Vasconcelos et al., 2014). In addition, *Leishmania* parasites and saliva induce an inflammatory response initiated by an influx of leukocytes to the feeding site (Kamhawi et al., 2000; Silva et al., 2005; Teixeira et al., 2005, 2014; Peters et al., 2009; Araújo-Santos et al., 2010, 2014; de Moura et al., 2010; Prates et al., 2012; Carregaro et al., 2013). Neutrophils are the first cells to rapidly mobilize and quickly internalize parasites at the site of infection (Peters et al., 2009). They modify the course of immunity and infection with different *Leishmania* species (McFarlane et al., 2008; Peters et al., 2009; Ritter et al., 2009; Charmoy et al., 2010; Ribeiro-Gomes et al., 2012; Sousa et al., 2014) and are able to promote activation and recruitment of different leukocytes (Ribeiro-Gomes et al., 2012; Schuster et al., 2013; Sousa et al., 2014). Since neutrophils are important elements during *Leishmania* infection, evaluation of Th17 immune responses has been considered relevant. Recent work has shown that cellular immunity generated by Th17 subsets, that have IL-17 as its main cytokine, display an important role in intracellular parasite infections (Lockhart et al., 2006; Meeks et al., 2009; Miyazaki et al., 2010; Erdmann et al., 2013). IL-17 induces iNOS activation, expression of granulocyte macrophage colony stimulating factor and several cytokines and chemokines. This results in the recruitment of leukocytes, especially neutrophils, creating a robust inflammatory infiltrate (Kolls and Linde, 2004). In leishmaniasis, IL-17 production could promote disease or protection depending on the *Leishmania* species and the context of infection (Gonçalves-de-Albuquerque et al., 2017). However, the possible role of sand fly saliva on IL-17 production during *Leishmania* infection remains unclear.

Following the initial influx of neutrophils to the site, a wave of monocytes, macrophages, and dendritic cells migrate to the infection site. *Leishmania* transitioning from infected neutrophils to macrophages and dendritic cells characterizes a later infection stage (Charmoy et al., 2010; Gonçalves et al., 2011; Petritus et al., 2012; Ribeiro-Gomes and Sacks, 2012; Ribeiro-Gomes et al., 2012). At the site of intradermal *Leishmania infantum* infection, infected neutrophils and macrophages colocalize in the cellular infiltrate 24 h after parasite inoculation (Thalhofer et al., 2011). The close interaction between different cells during the initial inflammatory infiltrate orchestrates the downstream immune response to the parasite. In fact, interaction between *L. major* infected neutrophils and dendritic cells impair dendritic cell function compromising *Leishmania* specific CD4^+^ T cells priming (Ribeiro-Gomes et al., 2012).

Sand fly saliva is also capable of inducing neutrophil and macrophage recruitment and modulating their function (Silva et al., 2005; Teixeira et al., 2005, 2014; Araújo-Santos et al., 2010; de Moura et al., 2010; Prates et al., 2012; Carregaro et al., 2013; Tavares et al., 2014). For instance, neutrophil and macrophage activity is impaired in the presence of *Lutzomyia longipalpis* saliva resulting in cell apoptosis, production of PGE_2_ and LTB_4_ promoting increased parasite survival (Araújo-Santos et al., 2010, 2014; Prates et al., 2011). Saliva from different species of sand flies affects macrophage function, resulting in modulation of IL-10 and NO production (Waitumbi and Warburg, 1998; Zer et al., 2001; Norsworthy et al., 2004). However, despite strong evidence of the immunomodulatory effects on different subsets of leukocytes, the impact of sand fly saliva on the interaction between different host cells during *Leishmania* infection have not been investigated.

The aim of this study was to further characterize the modulatory effect of *Lu. longipalpis* salivary gland sonicate (SGS) in the presence of *L. infantum* using an *in vitro* priming system and the consequences on neutrophil and macrophage infection during the initial stages. We demonstrated that *Lu. longipalpis* SGS display a selective action on CD4^+^ T cells modulating production of cytokines, increasing IL17-related neutrophil recruitment. Infection of human neutrophils in the presence of *Lu. longipalpis* SGS contributes to neutrophil apoptosis resulting in increased parasite viability. Additionally, infection of neutrophils in the presence of *Lu. longipalpis* SGS resulted in release of PGE_2_ and TGF-β and increased macrophage parasite loads. These findings indicate that *Lu. longipalpis* SGS is an important factor facilitating *Leishmania* survival representing an important mechanism of infection establishment.

## Materials and Methods

### Sand Flies and Preparation of Salivary Glands

*Lu. longipalpis* sand flies (Cavunge strain) were reared at Laboratório de Imunoparasitologia (IGM) using a mixture of rabbit feces and rabbit ration as larval food. Sand fly colonies were maintained at 26°C. Salivary gland sonicate (SGS) was obtained from 5 to 7-day-old laboratory-bred *Lu. longipalpis* females. Salivary glands were dissected, placed in endotoxin-free PBS on ice, and stored at -70°C. Immediately before use, glands were sonicated and centrifuged at 10,000 × *g* for 2 min, and the supernatant was used for the studies. The level of LPS contamination of SGS preparations was determined using the LAL Chromogenic Kit (QCL-1000, Lonza Bioscience, United States). Results detected negligible levels of endotoxin in the salivary gland supernatants. Experimental procedures used an amount equivalent to two pairs of SGS/well, representing ∼2.0 μg of protein (Prates et al., 2008).

### *Leishmania* Parasites

*Leishmania infantum* (MCAN/BR/89/BA262) promastigotes were cultured at 25°C in Schneider’s insect medium (Sigma Chemical Co., United States), supplemented with 10% heat-inactivated fetal bovine serum (Gibco, United States), L-glutamine (2 mM), penicillin (100 U/ml), and streptomycin (100 mg/ml) (Invitrogen, United States) at 23°C for 5–7 days when the parasites reached the stationary-phase.

### Ethics Statement

This study was approved by the Research and Ethics Comitee of FIOCRUZ – Bahia. All healthy blood donors had given written, informed consent.

### Isolation of PBMC

Human blood was obtained from healthy donors from Hemocentro do Estado da Bahia residing in a non-endemic area for Visceral Leishmaniasis (Salvador, Bahia, Brazil). PBMC were isolated by Ficoll-Hypaque (Sigma-Aldrich, United States) density gradient separation and cultured in RPMI-1640 supplemented with L-glutamine (2 mM), penicillin (100 U/ml), streptomycin (100 μg/ ml) (Invitrogen, United States), and heat inactivated AB Rh^+^ human serum (Sigma Chemical Co., United States).

### PBMC Culture and Flow Cytometry

PBMC were placed in 48-well plates (Corning Incorporated Life Sciences, United States) at a concentration of 1.5 × 10^6^ cells/ml and unstimulated or stimulated with *Lu. longipalpis* SGS (2.0 pair/well), *L. infantum* (2 cells:1parasite), or *Lu. longipalpis* SGS plus *L. infantum* for 96 h at 37°C, 5% CO_2_ (Clarêncio et al., 2006; Oliveira et al., 2012; Santos et al., 2013). During the last 4 h of culture, brefeldin A (BD Biosciences, United States) was added to the cultures. The cells were fixed and permeabilized using cytofix/cytoperm solution (BD Biosciences, United States) and stained for 30 min at 4°C using monoclonal antibodies directly conjugated to fluorochromes, against CD4 FITC (RPA-T4), CD8 PeCy5 (RPA-T8), IFN-γ PE (4S.B3), TNF-α PE (Mab11), IL-10 PE (JE53-19F1), IL-17 PE (SCPL1362). FITC, PE, and PeCy5-labeled immunoglobulin control antibodies were included in all experiments. In all cases 100,000 events/sample for PBMC were acquired in a FACSort flow cytometer (BD Immunocytometry, United States). All data were analyzed using FlowJo software.

### IL-17 Measurement

IL-17 levels were determined using the DuoSet^®^ ELISA Development System (R&D Systems, United States) according to manufacturer’s protocol.

### Isolation and Infection of Human Neutrophils

Neutrophils were isolated by centrifugation using PMN medium according to manufacturer’s instructions (Robbins Scientific Corporation, United States). Briefly, blood was centrifuged for 30 min at 300 g at room temperature. Neutrophils were collected and washed three times at room temperature at 200 g and then cultured in RPMI-1640 medium supplemented with 10% Fetal Bovine Serum (Gibco, United States), 2 mM L-glutamine, 100 U/ml penicillin, and 100 μg/ml streptomycin (Invitrogen, United States) and infected with stationary-phase *L. infantum* parasites (1 cell:5 *L. infantum*) in 48 well plates, in the presence or not of *Lu. longipalpis* SGS (two pairs/ well) for 3 h at 37°C under 5% CO_2_ (Prates et al., 2011).

### Chemotactic Assays

Isolated PBMCs were incubated with medium (RPMI-1640), *Lu. longipalpis* SGS, *L. infantum* or *L. infantum* plus *Lu. longipalpis* SGS, as described above. After 96 h, culture supernatants from each experimental group were harvested and incubated with anti-hIL-17 for 30 min (AF-317-NA, R&D Systems; 20 μg/ml) to neutralize the IL-17 secreted before chemotactic assay. After that, these supernatants pretreated or not (control) with anti-hIL-17 were added to the bottom wells of a 96-well chemotaxis microplate ChemoTx system (Neuro Probe, United States) as previously described (Prates et al., 2011; Beerli et al., 2014). Then, human neutrophils isolated by centrifugation using PMN (Robbins Scientific Corporation, United States), as described above, were resuspended in RPMI-1640 medium before being added to the top wells (10^5^ cells/well) and incubated for 1 h at 37°C under 5% CO2. Following incubation, cells that migrated to the bottom wells were counted using a hemocytometer. Neutrophil migration toward RPMI-1640 medium alone (random chemotaxis) was used as a negative control. The chemotaxis index was calculated as the ratio of the number of migrated cells toward supernatants taken from PBMC unstimulated or stimulated with *Lu. longipalpis* SGS, *L. infantum*, or *Lu. longipalpis* SGS plus *L. infantum* followed or not with anti-hIL-17 pretreatment to the number of cells that migrated to RPMI-1640 medium alone.

### Neutrophil Apoptosis Assay

Neutrophils were cultured in 200 μl in RPMI-1640 medium, supplemented with 2 mM L-glutamine, 100 U/ml penicillin, and 100 μg/ml streptomycin (Invitrogen, United States) in 96-well plates (Nunc, Denmark) in the presence of *Lu. longipalpis* SGS (2.0 pair/well). Three hours after infection neutrophil apoptosis was assessed with annexin-V-FITC (BD Biosciences, United States) in combination with PI PE (BD Biosciences, United States) nuclear dye using a FACSort flow cytometer (BD Immunocytometry, United States). Data was analyzed using FlowJo software.

### Assessment of Intracellular Load of *L. infantum*

Human neutrophils were treated or not with *Lu. longipalpis* SGS and infected with stationary-phase *L. infantum* (1 cell:5 parasites) for 3 h. After washing to remove non-internalized parasites, RPMI-1640 media was replaced for Schneider’s insect medium (Sigma Chemical Co., United States), supplemented with 10% heat-inactivated fetal bovine serum (Gibco, United States), L-glutamine (2 mM), penicillin (100 U/ml), streptomycin (100 mg/ml) (Invitrogen, United States) at 23°C for 24 h. Subsequently, the number of viable *L. infantum* were determined by counting live promastigotes using a Neubauer chamber.

### Differentiation of Macrophages

Monocytes were obtained from adherence for 30 min of total PBMC (3 × 10^6^/well) in 24 well plates containing sterile glass coverslips at the bottom. Non adherent cells were removed and monocytes were cultured in RPMI-1640 supplemented with 10% heat-inactivated fetal bovine serum (Gibco, United States), L-glutamine (2 mM), penicillin (100 U/ml), streptomycin (100 mg/ml) (Invitrogen, United States) for 7 days for macrophage differentiation (Vinhas et al., 2007).

### Co-culture of Autologous Neutrophils and Macrophages

On day 7 of macrophage culture, autologous *L. infantum-*infected neutrophils were added to the respective autologous macrophages (1 macrophage to 5 neutrophils ratio) and incubated for 24 h. Macrophages directly infected with stationary-phase *L. infantum* (1 cell:5 *L. infantum* ratio) were used as a control. After infection neutrophils and macrophages were washed with sterile saline to eliminate parasites that were not internalized and cultured with RPMI-1640 medium, supplemented with 1% Nutridoma-SP, 2 mM L-glutamine, 100 U/ml penicillin, and 100 μg/ml streptomycin (Invitrogen, United States; Afonso et al., 2008). Glass coverslips were harvested, fixed with ethanol, and stained with hematoxylin-eosin (HE). Intracellular amastigotes were counted in 200 macrophages. Results are shown as infection index determined by the percentage of infected macrophages multiplied by the average number of amastigotes per macrophage.

### PGE_2_ and TGF-β Measurement

Six and twenty four hours post infection of macrophages co-cultured with autologous infected neutrophils, supernatant was harvested for measurement of PGE_2_ (Cayman Chemical, United States) and TGF-β (R&D, United States) by ELISA according to manufacturer’s instructions.

### Statistical Analysis

One-way ANOVA (Kruskal–Wallis) analysis with Dunn’s post-test was done to compare different groups. All tests were performed using Prism software (GraphPad Software, United States). The results were considered statistically significant when *p* < 0.05.

## Results

### Stimulation of Human PBMC With *L. infantum* and *Lu. longipalpis* SGS Is Able to Induce Expression of Cytokines by CD4^+^ T Lymphocytes

**FIGURE 1:**
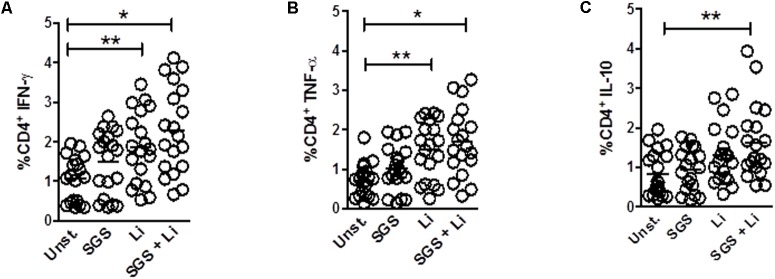
Frequency of cytokines expression on T CD4^+^ lymphocytes upon stimulation with *L. infantum* and *Lu. longipalpis* saliva. Peripheral blood mononuclear cells (PBMC) were stimulated with salivary gland sonicate from *Lu. longipalpis* (SGS), *Leishmania infantum* (Li), SGS plus *L. infantum* (SGS + Li), or remained unstimulated (Unst.) for 96 h, stained and analyzed by FACS for expression of **(A)** IFN-γ, **(B)** TNF-α or **(C)** IL-10 in the CD4^+^CD3^+^ lymphocyte population, *n* = 19 [^∗∗^*p* < 0.01 and ^∗^*p* < 0.05 between groups and unstimulated group (Unst)]. Experiments were repeated three times.

Initially, we investigated if stimulation with *L. infantum* in the presence of *Lu. longipalpis* SGS was able to modulate expression of cytokines by T cells obtained from healthy volunteers. We observed that expression of IFN-γ and TNF-α were significantly increased in CD4^+^ T cells when parasites were added alone or combined with *Lu. longipalpis* SGS. However, stimulation with SGS alone was not able to induce increased expression of IFN-γ and TNF-α (**Figures 1A,B**). A significant increase in IL-10 expression by CD4^+^ T cells was observed exclusively when *L. infantum* was incubated in the presence of SGS (**Figure 1C**). Interestingly, we did not observe any effect of *L. infantum* and *Lu. longipalpis* SGS on CD8^+^ T cells (data not shown).

### *Lu. longipalpis* SGS Is Able to Induce IL-17 Expression and Neutrophil Recruitment *in Vitro*

**FIGURE 2:**
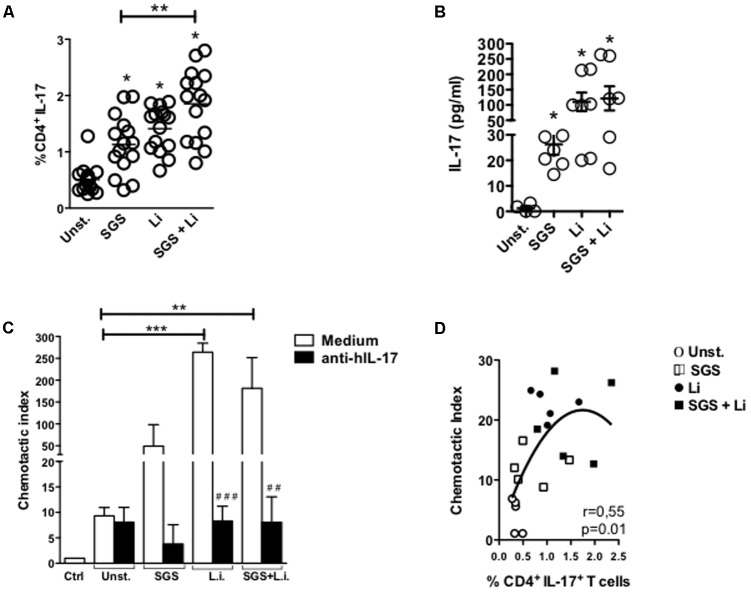
Stimulation with *L. infantum* and *Lu. longipalpis* saliva is able to increase IL-17 expression and neutrophil recruitment *in vitro*. Peripheral blood mononuclear cells (PBMC) were stimulated with salivary gland sonicate from *Lu. longipalpis* (SGS), *Leishmania infantum* (Li), or SGS plus *L. infantum* (SGS + Li) for 96 h, **(A)** stained and analyzed by FACS for expression of IL-17 in the CD4^+^CD3^+^ lymphocyte population, *n* = 14. **(B)** Production of IL-17 measured in the supernatant after 96 h, *n* = 7 [^∗∗^*p* < 0.01 and ^∗^*p* < 0.05 between groups and unstimulated group (Unst)]. **(C)** Neutrophils isolated from volunteers were allowed to migrate toward supernatants pretreated or not with anti-hIL-17 from PBMCs stimulated for 96 h with salivary gland sonicate from *Lu. longipalpis* (SGS), *Leishmania infantum* (Li), or SGS plus *L. infantum* (SGS + Li) The number of migrated neutrophils was determined and the chemotactic index was calculated [^∗∗^*p* < 0.01; ^∗∗∗^*p* < 0.001, between groups and unstimulated group (Unst.); ^##^*p* < 0.01; ^###^*p* < 0.001, compared with no anti-hIL-17-treated supernatants]. **(D)** Correlation between chemotactic index and expression of IL-17 by T CD4^+^ cells, *n* = 10. Experiments were repeated three times.

Incubation of PBMC with *Lu. longipalpis* SGS and *L. infantum* induced a significant increase in IL-17 expression on CD4^+^ T cells and production of IL-17 by stimulated PBMCs. Of note, SGS alone was able to induce increased IL-17 expression (**Figures 2A,B**). IL-17 performs an important function in neutrophil recruitment (Bedoya et al., 2013). Since Th17 cells induce recruitment of neutrophils to the site of inflammation, we also determined if the supernatants of PBMC culture stimulated with *L. infantum* in the presence or absence of SGS could promote recruitment of neutrophils in an *in vitro* cell migration assay. As shown in **Figure 2C**, supernatant from *L. infantum* and SGS plus *L. infantum* stimulated PBMC were able to induce neutrophil migration. The presence of SGS alone, was not able to induce a significant increase in neutrophil migration. In addition, incubation of supernatants with anti-hIL17 significantly reduced neutrophil recruitment (**Figure 2C**). Importantly, expression of IL-17 by CD4^+^T cells exhibited a direct positive correlation with neutrophil recruitment (**Figure 2D**).

### Increased Apoptosis and Parasite Viability in Neutrophils Infected in the Presence of *Lu. longipalpis* SGS

**FIGURE 3:**
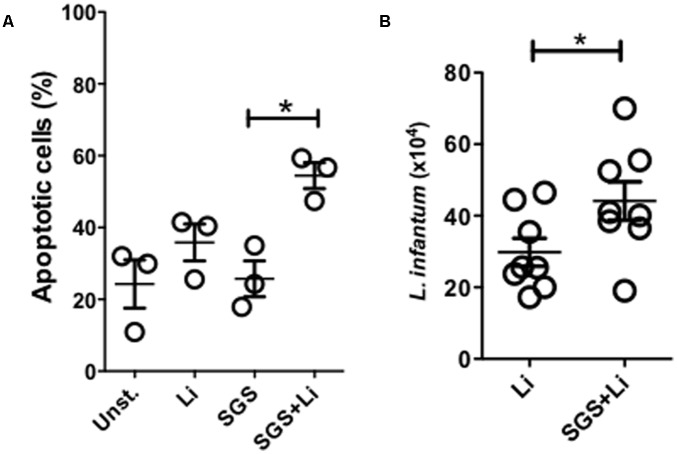
Neutrophils infected in the presence of *Lu. longipalpis* saliva display increased apoptosis and parasite viability. Neutrophils were infected with *L. infantum* in the presence (SGS+Li) or absence (Li) of salivary gland sonicate from *Lu. longipalpis* (SGS) for 3 hours. Following infection, neutrophils were washed to remove parasites that were not internalized. Neutrophil apoptosis was assessed by flow cytometry analysis after Annexin V x PI staining of *Leishmania*-infected neutrophils 3 h post-infection, *n* = 3 **(A)**. To evaluate parasite viability inside neutrophils, RPMI-1640 media was replaced by Schneider media and *Leishmania infantum* growth was assessed in both conditions after 24 h, *n* = 8 **(B)**, (^∗^*p* < 0.05). Experiments were repeated three times.

Previous work has shown that *Lu. longipalpis* saliva promotes apoptosis of inflammatory neutrophils promoting parasite growth (Prates et al., 2011). Here, we confirm these findings demonstrating that, as early as 3 h after *L. infantum* infection in the presence of *Lu. longipalpis* SGS, an increased number of apoptotic cells compared to the control or *L. infantum* without SGS (**Figure 3A**). Furthermore, infection of neutrophils in the presence of SGS also enhanced parasite viability inside these cells (**Figure 3B**).

### Infection of Neutrophils in the Presence of *Lu. longipalpis* SGS Increased Macrophage Infectivity *in Vitro*

**FIGURE 4:**
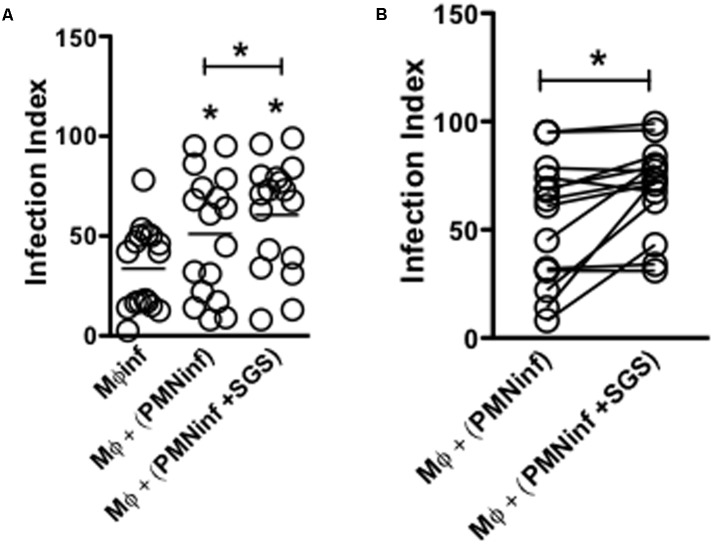
Infection of neutrophils in the presence of *Lu. longipalpis* saliva enhances macrophage infection. Neutrophils were infected with *L. infantum* in the presence (PMNinf + SGS) or absence (PMNinf) of salivary gland sonicate from *Lu. longipalpis* (SGS) for 3 h. Following infection, neutrophils were washed to remove parasites that were not internalized and then co-cultured with autologous macrophages for 24 h for **(A)** determination of macrophage infection index. Macrophages infected directly with *L. infantum* were used as a control (Mφinf). **(B)** Representation of macrophage infection index from each individual in the presence (PMNinf + SGS) or absence (PMNinf) of salivary gland sonicate from *Lu. longipalpis* (SGS) for 3 h, *n* = 16 (^∗∗^*p* < 0.01 and ^∗^*p* < 0.05 between groups). Experiments were repeated three times.

Macrophages are the preferential host cells for *Leishmania* that are recruited following the initial wave of neutrophil influx to the inflammatory site. However, the role of sand fly saliva on the interaction of neutrophils with macrophages during establishment of *L. infantum* infection is poorly understood. To assess the impact of *Lu. longipalpis* SGS on the transient passage of parasites in different cell populations, human neutrophils were initially infected with *L. infantum* in the presence or absence of SGS and were incubated with autologous macrophages. Macrophages that acquired parasites from neutrophils that were previously infected in the presence of *Lu. longipalpis* SGS showed a significant increase in infection (**Figure 4A**). Of note, we observed some degree of variation in macrophage infection levels (**Figure 4B**). Taken together, these results indicate that neutrophils infected in the presence of *Lu. longipalpis* SGS are able to more efficiently transfer infection to macrophages.

### *Leishmania infantum* Infected Human Neutrophils in the Presence of *Lu. longipalpis* SGS Increase PGE_2_ and TGF-β Production During Interaction With Macrophages

**FIGURE 5:**
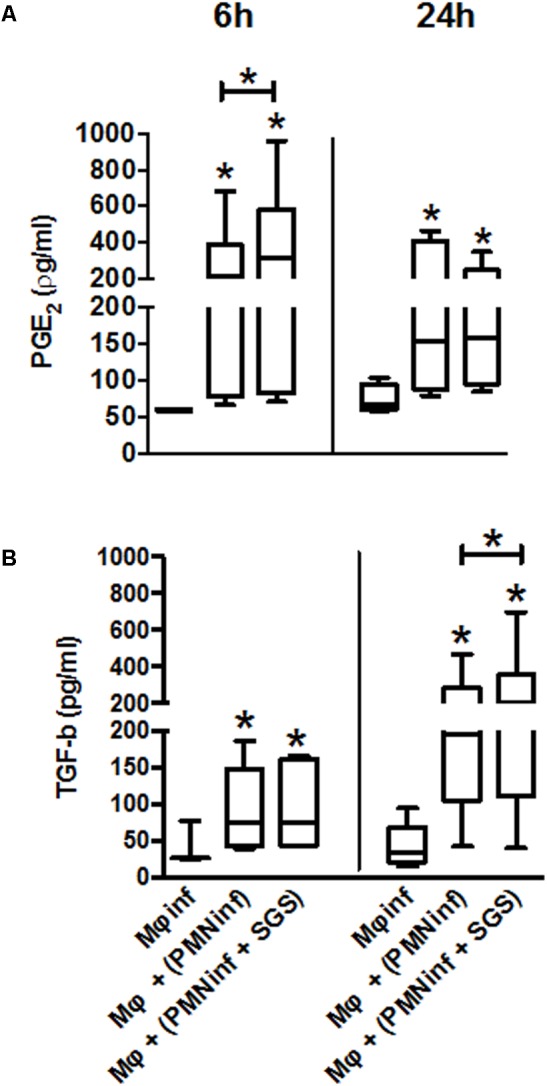
Production of PGE_2_ and TGF-β following co-culture of macrophages with neutrophils infected with *L. infantum* in the presence of *Lu. longipalpis* saliva. Neutrophils were infected with *L. infantum* in the presence (PMNinf + SGS) or absence (PMNinf) of salivary gland sonicate from *Lu. longipalpis* (SGS) for 3 h. Following infection, neutrophils were washed to remove parasites that were not internalized and then co-cultured with autologous macrophages for 24 h. Macrophages infected directly with *L. infantum* were used as a control (Mφinf). Supernatants were collected after 6 h and 24 h to determine **(A)** PGE_2_ and **(B)** TGF-β levels, respectively. Each bar represents median, quartile, and range, *n* = 18 [^∗∗^*p* < 0.01 and ^∗^*p* < 0.05 between groups and uninfected macrophages (Mϕ)]. Experiments were repeated three times.

As early as 6 h after infection, a significant increase in PGE_2_ and TGF-β production was observed when macrophages acquire *Leishmania* from infected neutrophils compared to uninfected macrophages. Interestingly, when neutrophils were infected in the presence of SGS, a dramatic increase of PGE_2_ (6 h) and TGF-β (24 h) production is observed (**Figures 5A,B**), suggesting that infection of neutrophils in the presence of *Lu. longipalpis* SGS contribute to the release of anti-inflammatory mediators.

## Discussion

The present study provides further evidence that *Lu. longipalpis* saliva plays an important role on the different steps of the inflammatory response initiated after transmission. We found that *Lu. longipalpis* SGS promote neutrophil recruitment and apoptosis. As a consequence, direct and indirect infection of neutrophils and autologous macrophages, respectively, are enhanced in the presence of saliva.

To explore the early immune events against *Leishmania* infection, *in vitro* models have been developed using human naïve T cells. Results have shown that *in vitro* cell priming systems can be used to delineate immune responses against *Leishmania* after *in vivo* infection (Russo et al., 1998, 1999; Brodskyn et al., 2001; Pompeu et al., 2001). Initially, we observed that stimulation of PBMCs obtained from healthy volunteers with *L. infantum* in the presence of *Lu. longipalpis* SGS modulate cytokine production by CD4^+^ but not CD8^+^ T cells. Importantly, the immunomodulatory effect of *L. infantum* and SGS on CD4^+^ T lymphocytes observed here is probably dependent on antigen presenting cells present in the PBMC cultures that will need further characterization. This finding adds evidence that arthropod saliva is able to interact and modulate subpopulations of T cells. Recently, it was shown that as early as 6 h after bites from *Phlebotomus duboscqi* infected with *L. major* CD4^+^ T lymphocytes are recruited to the bite site in naïve animals (Teixeira et al., 2014). This effect on lymphocytes has also been demonstrated with saliva from other arthropod vectors. A molecule identified in the saliva from the tick *Ixodes scapularis*, Salp15, is able to bind to CD4^+^ T cells inhibiting activation (Garg et al., 2006). Saliva from *Aedes aegypti* acts selectively on naïve T cells, but not memory T cells, inhibiting their proliferation (Bizzarro et al., 2013).

From the cytokines evaluated, IL-17 was the only one increased by the presence of *Lu. longipalpis* SGS alone displaying an enhanced effect when incubated with *L. infantum*. Interestingly, enhanced expression of IFN-γ and TNF-α was observed following *L. infantum* but were not altered by the presence of saliva. Although an increased IL-17 expression by CD4^+^ T cells was detected, we cannot exclude the possibility of other cells, such as NKT or γδ T cells, being able to secrete IL-17 at the inflammatory site (Shibata et al., 2007, 2011; Korn et al., 2009). IL-17 production by Th17 cells has been associated with inflammation and autoimmune diseases (Pitta et al., 2009; Shabgah et al., 2014; Astry et al., 2015). Excessive IL-17 production influence disease progression by regulating neutrophil recruitment in susceptible BALB/c mice during *L. major* infection (Lopez Kostka et al., 2009; Gonzalez-Lombana et al., 2013). In visceral leishmaniasis, it was shown that *L. donovani* promote IL-17 and IL-22 production by T CD4^+^ cells from healthy volunteers and patients. IL-17 production was strongly associated with protection demonstrated in a group of individuals residing in an endemic area in Sudan (Pitta et al., 2009). Similar findings were recently reported in dogs naturally infected with *L. infantum* showing that increased expression of Th1 cytokines and IL-17A play a protective role controlling parasite growth in asymptomatic animals (Nascimento et al., 2015). Moreover, development of a Th17 immune response and neutrophil recruitment through activation of 5-lipoxygenase and production of LTB_4_ contributed to control of *L. infantum* infection (Sacramento et al., 2014). On the other hand, in mucosal leishmaniasis, IL-17 was detected in lesions obtained from patients. The presence of a Th17 immune response was also accompanied by an intense infiltration of neutrophils that correlated with tissue damage (Boaventura et al., 2010). Thus, although the role of IL-17 in leishmaniasis differs between clinical forms and host species, its participation in the effector response during infection is unquestionable (Gonçalves-de-Albuquerque et al., 2017). This result was reinforced by pretreatment with anti-hIL17 antibody that significantly reduced neutrophil recruitment. Although IL-17 does not induce cellular recruitment directly, different chemokines are produced during a Th17 immune response leading to chemotaxis of neutrophils (Laan et al., 1999). Chemokines directly responsible for neutrophil recruitment observed following *Lu. longipalpis* SGS stimulation still need to be investigated. Additionally, production of IL-17 was accompanied by a significantly increased IL-10 expression by CD4^+^ T cells only observed when *Lu. longipalpis* SGS was added in the presence of *L. infantum*. In fact, saliva from *Lu. longipalpis* enhance *L. amazonensis* infectivity by stimulating IL-10 production in macrophages and T cells (Norsworthy et al., 2004). Production of IL-10 could play an important immunomodulatory role possibly preventing the uncontrolled and polarized inflammatory response induced by IL-17.

A rapid mobilization of neutrophils is one of the first events triggered after *Leishmania* transmission (Peters et al., 2009). Different species of *Leishmania* are known to exploit neutrophil function as an escape mechanism (Afonso et al., 2008; Gueirard et al., 2008; de Souza Carmo et al., 2010). We have previously demonstrated that *Lu. longipalpis* saliva is able to induce neutrophil apoptosis promoting parasite survival (Prates et al., 2011). Here, we demonstrate an increased frequency of apoptotic neutrophils confirming these findings. This is an important step in the dynamics of inflammation resolution since neutrophil recruitment is naturally followed by a wave of macrophages that are attracted to remove apoptotic neutrophils. Inevitably, the two cell types will interact during this process. Macrophages are responsible for the uptake and removal of apoptotic cells at the inflammatory site getting in contact with infected neutrophils (Ribeiro-Gomes et al., 2007; Afonso et al., 2008; de Souza Carmo et al., 2010). Apoptotic neutrophils provide a safe haven for parasite entry into macrophages (de Souza Carmo et al., 2010). This has been reported as an important step for establishment of *Leishmania* infection in the host (Laan et al., 1999; Van Zandbergen et al., 2004; Ribeiro-Gomes et al., 2012; Sacramento et al., 2014). Our findings also demonstrated that, in the presence of *Lu. longipalpis* saliva, an increased number of parasites was observed in neutrophils. Therefore, the increase of parasites observed in macrophages cultured with previously infected neutrophils could be due to the presence of apoptotic cells promoting an anti-inflammatory environment for *L. infantum* proliferation or to a higher number of parasites inside neutrophils infected in the presence of saliva.

We have recently shown that inoculation of *Lu. longipalpis* saliva with *L. infantum* results in induction of PGE_2_ production by macrophages contributing for an increased parasite burden (Araújo-Santos et al., 2014). Here, our results reinforce this finding showing that infection of neutrophils with *L. infantum* in the presence of *Lu. longipalpis* SGS induces an initial increase in PGE_2_ followed by TGF-β production. Suppression of pro-inflammatory cytokines through production of TGF-β, PGE_2_ and PAF has been previously described following uptake of apoptotic neutrophils by macrophages during the inflammatory process (Fadok et al., 1998). Similar results have been demonstrated during *L. amazonensis* infection where uptake of apoptotic human neutrophils resulted in increased parasite burden dependent on PGE_2_ and TGF-β production (Afonso et al., 2008). *Leishmania major* infected neutrophils can also transit to other cells, such as dendritic cells and monocytes. In fact, Ribeiro-Gomes et al. (2012) have demonstrated that *L. major* infected neutrophils interact with dendritic cells suppressing anti-*Leishmania* adaptive immune responses .

Taken together, our results demonstrate that *Lu. longipalpis* saliva contributes to the immunomodulatory mechanisms induced by *L. infantum*. Production of IL-17, induced by *Lu. longipalpis* saliva and the parasite, results in neutrophil recruitment and apoptosis creating an environment that benefit parasite growth. The presence of anti-inflammatory mediators increases the chances of macrophage infection at a later stage of the inflammatory response. To our knowledge, this is the first description that *Lu. longipalpis* saliva induces increased IL-17 expression by CD4^+^T cells contributing to the interaction between neutrophils and macrophages and *L. infantum* survival. Our results reinforce the importance of investigating the role of sand fly saliva and the immunologic consequences on the different steps of the inflammatory response at the site of primary contact with *Leishmania*.

## Author Contributions

All authors conceived and designed the study and contributed to data analysis. CT, CS, DP, RS, TA-S, and SS-N performed the laboratory work. CT, CS, DP, RS, TA-S, VB, MB-N, and CB contributed to writing the manuscript and gave final approval for publication.

## Conflict of Interest Statement

The authors declare that the research was conducted in the absence of any commercial or financial relationships that could be construed as a potential conflict of interest.
